# LED-CT Scan for pH Distribution on a Cross-Section of Cell Culture Medium

**DOI:** 10.3390/s18010191

**Published:** 2018-01-11

**Authors:** Nobuya Higashino, Toshio Takayama, Hiroaki Ito, Mitsuhiro Horade, Yasutaka Yamaguchi, Chia-Hung Dylan Tsai, Makoto Kaneko

**Affiliations:** 1 Department of Mechanical Engineering, Osaka University, 2-1 Yamadaoka, Suita, Osaka 565-0871, Japan; higashino@hh.mech.eng.osaka-u.ac.jp (N.H.); ito@hh.mech.eng.osaka-u.ac.jp (H.I.); horade@mech.eng.osaka-u.ac.jp (M.H.); yamaguchi@mech.eng.osaka-u.ac.jp (Y.Y.); mk@mech.eng.osaka-u.ac.jp (M.K.); 2 Department of Mechanical Engineering, National Chiao Tung University, 1001 University Road, Hsinchu 300, Taiwan; dylantsai@nctu.edu.tw

**Keywords:** pH measurement, cell culture, non-contact measurement

## Abstract

In cell culture, the pH of the culture medium is one of the most important conditions. However, the culture medium may have non-uniform pH distribution due to activities of cells and changes in the environment. Although it is possible to measure the pH distribution with an existing pH meter using distributed electrodes, the method involves direct contact with the medium and would greatly increase the risk of contamination. Here in this paper, we propose a computed tomography (CT) scan for measuring pH distribution using the color change of phenol red with a light-emitting diode (LED) light source. Using the principle of CT scan, we can measure pH distribution without contacting culture medium, and thus, decrease the risk of contamination. We have developed the device with a LED, an array of photo receivers and a rotation mechanism. The system is firstly calibrated with different shapes of wooden objects that do not pass light, we succeeded in obtaining their 3D topographies. The system was also used for measuring a culture medium with two different pH values, it was possible to obtain a pH distribution that clearly shows the boundary.

## 1. Introduction

Our previous study shows that a cellular tissue cultured under periodic pressure would gain a greater elasticity than the ones without such pressurized environment [1,2]. However, the change of such a pressurized culture would also change the amount of CO_2_ dissolved in the culture medium, and as a result, it may generate an undesired gradient of pH in the cell culture medium. The pH of the cell culture medium is an important condition for cell culturing [3,4,5,6], and this gradient of pH can affect to growth of the cells. In order to precisely control the culture conditions, it is required to measure the distribution of pH inside the cell culture medium, and is also the motivation of this work.

To observe the time-lapse transition of pH of the cell culture medium, methods that obtain the pH quantitatively by measuring the color change of Phenol red added in the cell culture medium have been proposed [7,8,9,10,11,12]. In these methods, the sensor probe does not contact with the cell culture medium, thus the measurement would be contamination free. However, it cannot measure the distribution of the pH because it measures pH assuming that the pH of the culture medium is uniform in the whole area.

**Figure 1:**
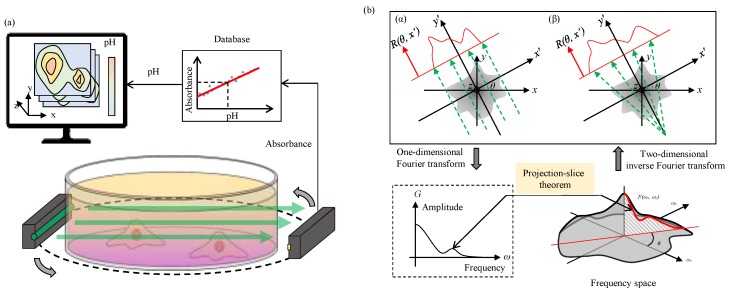
A method to measure pH distribution by computed tomography (CT) scan. (**a**) The concept of the system and (**b**) the algorithm of CT scan using the projection-slice theorem by (α) parallel beam or (β) fan beam.

In this paper, we propose a novel method to measure the distribution of the pH in the cell culture medium by using the principle of computed tomography (CT) scan. Depend on pH, phenol red can change its absorbance of green wavelength [13]. Thus, in the proposed method, a green light source and an array of photodetector are set around the dish to measure the absorbance of green light from various aspects as shown in Figure 1a. By using CT scan [14,15,16,17] and database of the relation between the absorbance and pH, we can obtain the distribution of pH. The goal of this study is to measure the distribution of pH in the culture medium in a clear container.

The rest of the paper is organized as follows. In Section 2, we briefly explain the basic principle of CT scan and the construction of the experimental device. In Section 3, we first measure opaque objects to obtain its contours for confirming the resolution of the developed device, and then we measure the distribution of absorbance in the colored objects. In Section 4 we discuss the cause of unmeasurable areas, and the conclusions are summarized in Section 5.

## 2. Materials and Methods

### 2.1. Basic Principle of CT Scan

This section briefly explains basic principle of CT scan by using parallel scan lines shown in Figure 1. Assume *f*(*x*, *y*) as a distribution of absorbance on *xy* plane. *x*′*y*′ plane has an angle of *θ* with respect to *xy* plane on *z* axis. When parallel light is irradiated along *y*′, the projected distribution of absorbance along *x*′ can be calculated as

(1) $$R(\theta ,x\prime )=\int_{-\infty }^{\infty }f(x,y)dy\prime$$

For simplification of later analysis, we let G(θ,ω) be Fourier transformed *R* about *x*′.

(2) $$\begin{array}{cc} G(\theta ,\omega ) & =\int_{-\infty }^{\infty }R(\theta ,x^{\prime })e^{-i\omega x^{\prime }}dx^{\prime }\\ & =\int_{-\infty }^{\infty }\int_{-\infty }^{\infty }e^{-i\omega x^{\prime }}f(x,y)dy^{\prime }dx^{\prime }\\ & =\int_{-\infty }^{\infty }\int_{-\infty }^{\infty }e^{-i\omega (x\cos\theta +y\sin\theta )}f(x,y)dxdy\\ & =\int_{-\infty }^{\infty }\int_{-\infty }^{\infty }e^{-i(ux+vy)}f(x,y)dxdy\\ & \left(u=\omega \cos\theta ,v=\omega \sin\theta \right) \end{array}$$

Assume *F*(*u*, *v*) as a two dimensional Fourier-transformation of *f*(*x*, *y*).

(3) $$F(u,v)=\int_{-\infty }^{\infty }\int_{-\infty }^{\infty }e^{-i(ux+vy)}f(x,y)dxdy$$

Then *f*(*x*, *y*) can be regenerated by inverse Fourier-transformation of *F*(*u*, *v*).

(4) $$f(x,y)={\left(1/2\pi \right)}^{2}\int_{-\infty }^{\infty }\int_{-\infty }^{\infty }e^{i(ux+vy)}F(u,v)dudv$$

From u=ωcosθ, v=ωsinθ, a change of variables is applied to the double integral.

(5) $$\int_{-\infty }^{\infty }\int_{-\infty }^{\infty }dudv=\int_{0}^{2\pi }d\theta \int_{0}^{\infty }\omega d\omega$$

By substituting Equation (5) into Equation (4), we can solve *f*(*x*, *y*) as

(6) $$\begin{array}{cc} f(x,y) & ={\left(1/2\pi \right)}^{2}\int_{-\infty }^{\infty }\int_{-\infty }^{\infty }e^{i\omega (x\cos\theta +y\sin\theta )}F(\omega \cos\theta ,\omega \sin\theta )\omega d\omega d\theta \\ & =\frac{1}{2\pi }\int_{0}^{2\pi }\left(\frac{1}{2\pi }\int_{0}^{\infty }e^{i\omega (x\cos\theta +y\sin\theta )}G(\theta ,\omega )\omega d\omega \right)d\theta \end{array}$$

According to Equation (6), the pH distribution, which is represented by the absorbance of green light *f*(*x*, *y*), can be determined from the scan result *G*. However, parallel light source requires a high density light array or a mechanism to move light source along *x*′ axis. The former is difficult to make and the latter makes the device complicated. Therefore, we use a point light source with radial scan lines, which is also known as “Fan Beam” in CT technology, and is illustrated in Figure 1(bβ). There are different methods to resolve the Fan Beam projections for reconstructing the CT image [18]. In this paper, we utilize an additional transformation matrix *h* to convert Equation (1) for Fan-Beam projection and the Equation (1) becomes

(7) $$R(\theta ,x^{\prime })=\int_{-\infty }^{\infty }hf(x,y)dy^{\prime }$$

The calculation for determining the distribution of pH in the experiments from the scanned result to will be performed using Equation (6) with replacement of Equation (1) with Equation (7).

### 2.2. Experimental Procedure

A light absorbance *A* is defined as

(8) $$A=-\log_{10}\frac{I}{I_{0}}$$

where *I*_0_ and *I* are the intensities of the incident light and transmitted light, respectively. The follows demonstrate a step-by-step procedure of generating distribution of absorbance image in *xy* plane.

1. The intensity of the light along *x*′ axis is measured without placing any object. This data is used as the background for calibration and is represented by *I*_0_(*x*′).
2. A target object, such as a container with different pH liquid, is placed inside the sensing area, and the intensities along the *x*′ axis at different angles *θ* are measured. The data is denoted as *I*(*θ*, *x*′).
3. The absorbance data *R*(*θ*, *x*′) is processed by Equation (7).
4. Fourier-transformation of *R*(*θ*, *x*′) is applied for obtaining *G*(*θ*, *ω*) with Equations (2) and (7).
5. The distribution of absorbance *f*(*x*, *y*) is obtained by Equation (6). We use “ifanbeam” function in MatLab to regenerate *f*(*x*, *y*) for the data analysis.

### 2.3. Experimental Setup

**Figure 2:**
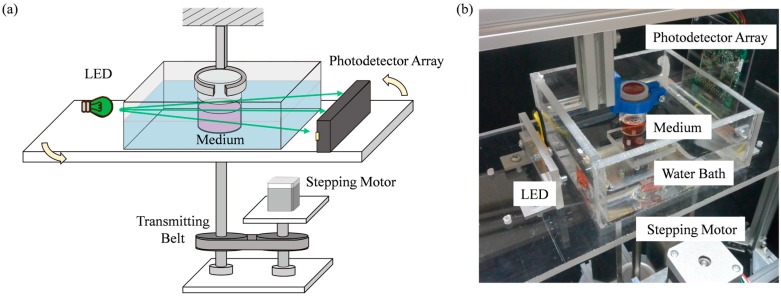
The experimental device. (**a**) A schematic drawing of the system and (**b**) a photo of the system.

Figure 2a,b show a schematic drawing and an actual photo of the experimental device, respectively. The device consists of a green LED light source (GM5GC01250AC, Sharp Corporation, Osaka, Japan), a photodetector array (S11865-64, Hamamatsu Photonics K.K., Shizuoka, Japan), a water bath, and a stepping motor (ST-42BYH1004, Mercury Motor, Shenzhen, China). The reason of choosing a green LED as the light source is explained in Appendix A. The photodetector array has 64 photo diodes evenly distributed along a length of 5 cm. The LED, the photodetector array and the water bath are fixed on the same board, and the board is rotated around a measuring object by a stepping motor through a transmitting belt as shown in Figure 2a. A bottle filled with medium is used as a target object. The type of medium used in the experiment is phenol red with the density of 0.0159 g/L which corresponds to Dulbecco’s Modified Eagle Medium (D5796, MilliporeSigma Canada Co., Oakville, ON, Canada). The bottle is located between the LED and the photodetector, and is hanged from the upper frame so that it does not rotate with the board. If the clear container rotates itself, the fluid in it would be mixed and the distribution would be changed. To keep the medium in stationary, the bottle is fixed in the absolute coordinate system as shown in Figure 2.

**Figure 3:**
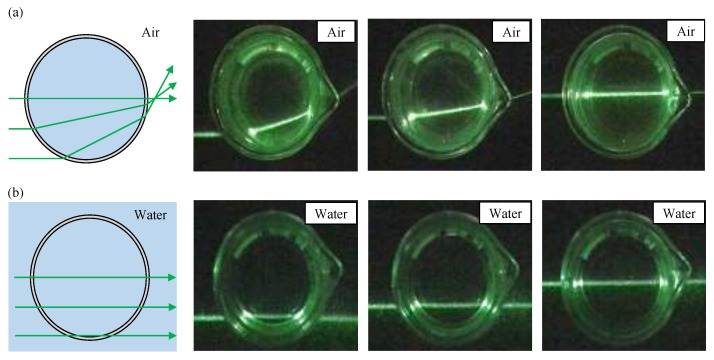
The path ways of parallel laser beams pass through a glass bottle. (**a**) The refraction of light without a water bath and (**b**) the refraction of light with a water bath.

The water bath is filled with water before the measurement. This is to suppress the effect coming from the refraction of the light when it passes through the bottle. Figure 3a shows the path ways of laser beams without water bath. The refraction of the light can be easily visualized. In Figure 3b the bottle is placed in the water bath, and it effectively reduce the refraction. In case of non-parallel laser beam, see Appendix B.

### 2.4. Response Speed

**Figure 4:**
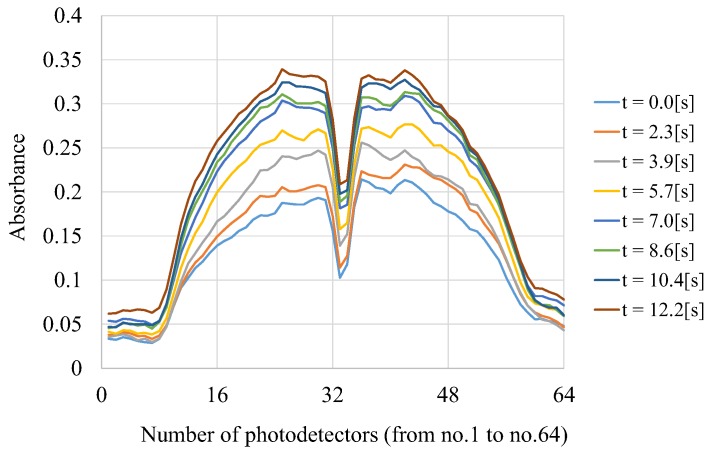
Change of the absorbance when the acidic culture medium is gradually changed to alkaline.

In order to confirm the response speed of the proposed LED-CT pH scanning, we conducted experiments measuring the pH change when the pH of the culture medium was gradually changed. Figure 4 is the results when alkaline culture medium was gradually added to acidic culture medium, and this measurement was performed from a fixed angle. The vertical and horizontal axes show the absorbance measured by developed device and the number (from no. 1 to no. 64) of photodetectors, respectively. From this graph, it can be seen that the absorbance of the culture medium gradually increases, which indicates the changes in pH.

## 3. Results

### 3.1. In Case of 100% Absorbance at the Surface

**Figure 5:**
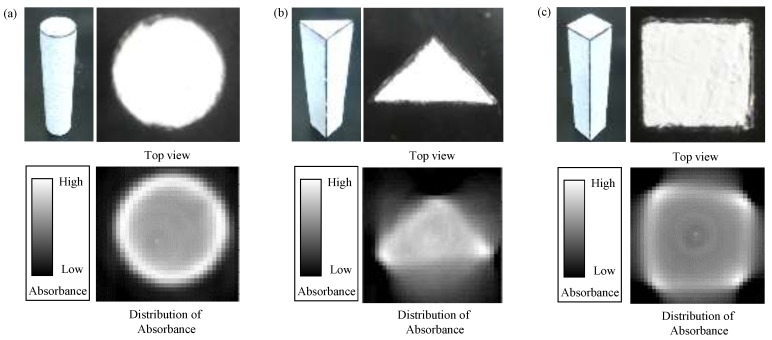
The experimental result of obtaining outlines of wooden objects. (**a**) A cylindrical column and (**b**) a triangular prism and (**c**) a quadrangular prism.

For observing the results under 100% absorbance at the surface, we made a non-transparent cylinder, a triangular prism and a quadrangular prism made of wood. Since light cannot be transmitted through these objects, it is considered that the absorbance is 100%, that is, no light can penetrate the object. The results of this experiment are shown in Figure 5. Under 100% observation at the surface, we resultantly obtain the outline of the sample as shown in Figure 5. The resolution of the obtained image is affected by the angular resolution of the rotating base as shown in Appendix C.

### 3.2. Application to pH Measurement

**Figure 6:**
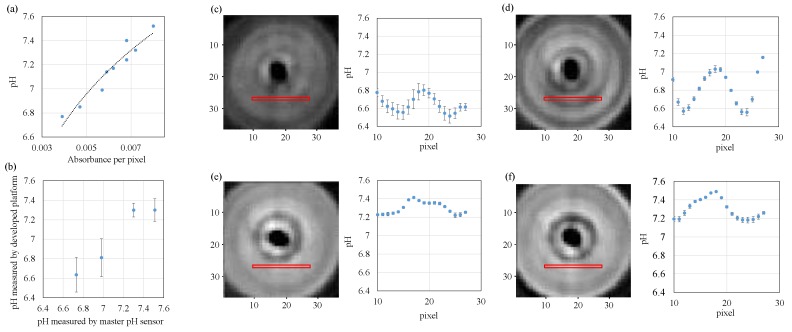
Experimental result of calculating pH from the obtained absorbance. (**a**) A database showing the relationship between absorbance and pH; (**b**) a result of mean values and standard deviations for each pH. Results of measurements on a sample with (**c**) pH 6.73; (**d**) pH 6.98 (**e**) pH 7.30 and (**f**) pH 7.51.

By utilizing the developed system, we apply it on pH measurements where one is single phase liquid sample and the other is liquid/liquid two-phase sample. Figure 6a shows the calibration data where horizontal and vertical axes show the absorbance per pixel and pH measured by a commercial sensor (SPM55, SANSYO Co., Tokyo, Japan), respectively. Sensitivity and repeatability of this system can be roughly evaluated by ΔpH/Δabsorbance and correlation coefficient *R*^2^, respectively. From Figure 6a, we can compute ΔpH/Δabsorbance ≈ 2.0 × 10^2^ and *R*^2^ ≈ 0.921. Figure 6b is the result of mean values and standard deviations for each pH by averaging all data, where the horizontal and vertical axes show pH measured by master pH sensor and the computed pH measured by the developed platform, respectively. Figure 6c–f are examples of pH measurement by using phenol red with different pH where Figure 6c–f are pH = 6.73, 6.98, 7.30 and 7.51, respectively. In order to suppress the influence of the reflection by the glass bottle, we subtract the measurement result of the bottle containing water from the measurement results in culture medium. Since the measurable pH range with this device depends on the color change range of phenol red (pH 6.8–8.4, #163-20623, Wako Pure Chemical Industries, Osaka, Japan), we chose the pH of “6.73”, “6.98”, “7.30” and “7.51” in experiments. The limits of pH sensing can be adjustable by different pH indicators. From Figure 6c–f, we can see the following statistic behaviors where the average of standard deviations are 0.154, 0.059, 0.033, 0.048 respectively, from which we can roughly evaluate the resolution of the sensing platform.

**Figure 7:**
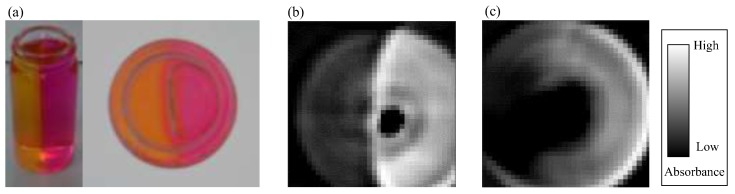
Experiment result on a sample with pH distribution. (**a**) An overview and a top view; (**b**) result when there is water around the sample and (**c**) when there is no water around the sample.

Figure 7a shows a photo of liquid/liquid two-pH sample where the right half and the left half are filled with gelatin with different color for each. The pH distribution measurement result of this sample is shown in Figure 7b. The green wavelength has a property that the absorbance is low in the yellow part which is acidic and the absorbance is high in the red part which is basic, which agrees with the experimental result. In addition, it is possible to accurately measure the rising of the vicinity of the bottle, which occurs when it is solidified with gelatin. This result suggests that non-contact pH distribution measurement of culture medium is possible by this method.

In addition, in order to confirm the effect of filling the surroundings of the bottle with water, the measurement result without water is shown in Figure 7c. Compared to Figure 7b, the pH boundary is not clear. From this result, it is found that the boundary between two samples with different pH can be clearly observed by filling the surroundings of the bottle with water. However, the sensor calibration range exceeds the full scale, we could not convert to pH as far as this experiment is concerned.

A clear observation in Figure 6 and Figure 7 is that there is a black area in the central part of the bottle. This black area corresponds to the region in Figure 4 where the absorbance is sharply down. The result that the absorbance for central part of the bottle is low means that the LED light pass through that area just like a transparent vessel.

## 4. Discussion

As for methods for measuring pH of culture medium, there are two methods where one is contact method such as pH electrodes [19] and the other is non-contact method [7,8,9,10,11,12]. Furthermore, there is a method capable of measuring 3D pH distribution [20]. The authors of [20] has discussed a similar approach in their work but their shape of the sample vessel is fixed only with polygonal shape to avoid light refraction. Circular shape which we are using for dish is the most popular for cell culturing. We would note that the principle we developed can be also applied for the dish with polygonal shape, which means we can apply for dishes with more general shape.

The strength point of the proposed method is that the developed system can be utilized even irrespective of the shape of dish, while the conventional works can not. The weakness point under the current version is that both sensitivity and resolution are not sufficient for practical use. We believe that this weakness can be cleared by using a laser instead of LED.

## 5. Conclusions

We proposed a method that can measure the pH distribution in culture medium in non-contact manner by using the principle of CT scan. We developed a sensor system consists of a green LED light source, a photodetector array, a water bath and a stepping motor for rotating both them around the medium bottle. By utilizing the developed system, we showed the relationship between pH measured by a commercial system and the absorbance obtained by the developed system. We also showed the sensitivity of ΔpH/Δabsorbance ≈ 1.91 × 10^2^ and the resolution with the correlation coefficient of *R*^2^ ≈ 0.921. We hope that both sensitivity and resolution will be improved by increasing the density of the photodetector array and the resolution in the base rotation.

## Figures and Tables

**Figure A1:**
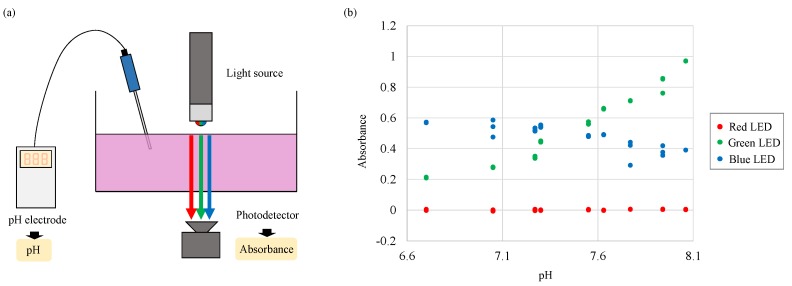
Experiments for the absorbance of lights in different pH solutions. (**a**) The experimental setup; (**b**) The result shows that the absorbance of the green light is affected by pH the most.

**Figure A2:**
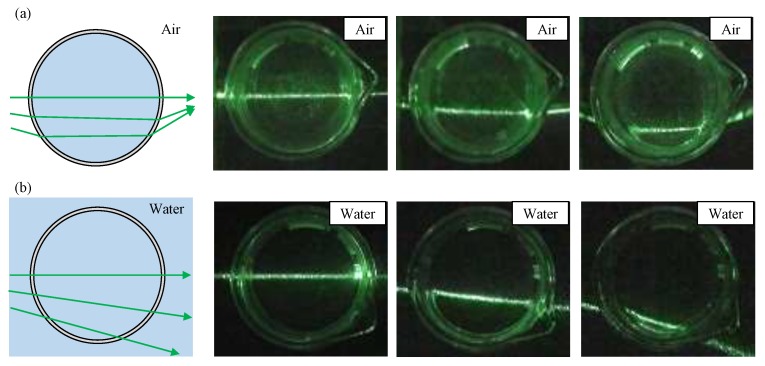
The path ways of non-parallel laser beams pass through a glass bottle. (**a**) The refraction of light without a water bath and (**b**) the refraction of light with a water bath.

**Figure A3:**
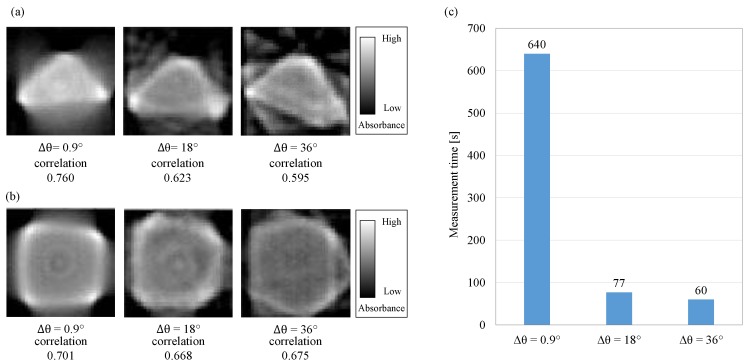
The outline when changing the resolution Δ*θ* = 0.9 °, 18 °, and 36 °. (**a**) A triangular prism; (**b**) a quadrangular prism and (**c**) the scanning time for recording.

## Appendix A

In this paper, we use a green LED as the light source of CT scan because the light absorbance differs the most in phenol red under different pH values. Figure A1 shows the absorbance of different colors of the light source. Figure A1a illustrates the experimental setup. We used one LED that contain three light colors, and they are red (*λ* = 630 nm), green (*λ* = 525 nm) and blue (*λ* = 465 nm), respectively. The photodetector measures its luminous intensity through uniform phenol red solutions with different pH, which is calibrated by a commercial pH meter (SANSYO Co., SPM55). Figure A1b shows that the red light is not absorbed by phenol red, the blue light is slightly affected by the difference of pH and the green light is affected the most by the difference of pH. As a result, we chose green LED as the light source in this paper.

## Appendix B

Figure A2 shows that the pass ways of non-parallel laser beam through a glass bottle where (a) and (b) are the case without a water bath and with a water bath, respectively.

## Appendix C

There are several factors to affect the resolution of the scanning system, such as angular resolution of the rotating base, where the LED for emission and the 64 photodetectors are mounted, the spread angle of light emission, the spatial interval between detectors, and so forth. Here, we focus on the angular resolution, whose minimum resolution is 0.9°, of the moving base because measurement time and cost of the device depend on angular resolution. Figure A3 shows the absorbance distribution for three different angular resolutions with Δ*θ* = 0.9°, 18°, and 36° from the left to the right, respectively. Figure A3a,b shows the results for two different shapes of column objects whose cross sections are triangular and square, as shown in Figure 5b,c, respectively. Figure A3c shows the time it takes for a scanning with Δ*θ* = 0.9°, 18°, and 36°. The scanning time is decreasing as Δ*θ* increases.

To quantify the goodness of the scanned result, we also calculate the pixel-by-pixel correlation between the experimental results and the ideal result. The ideal result is generated based on the dimension of the objects and is filled with white color as the reference. In Figure A3, we can see that the correlation reaches to its highest correlation numbers of 0.760, 0.701 under the finest angular resolution of Δ*θ* = 0.9°. The correlations changed drastically as Δ*θ* increases. The angular resolution of the scanning is critical for the spatial resolution of pH distribution.
